# Non-cardiogenic pulmonary oedema after neostigmine given for reversal: A report of two cases

**DOI:** 10.4103/0019-5049.68386

**Published:** 2010

**Authors:** Lalit Kumar Raiger, Udita Naithani, Bhavani S Vijay, Pradeep Gupta, Vaibhav Bhargava

**Affiliations:** Department of Anaesthesiology and Critical Care, R.N.T. Medical College and AG of Hospital, Udaipur - 313 001, Rajasthan, India

**Keywords:** Drug-related NCPE, neostigmine, reversal, non-cardiogenic pulmonary oedema

## Abstract

Non-cardiogenic pulmonary oedema (NCPE) is a clinical syndrome characterized by simultaneous presence of severe hypoxemia, bilateral alveolar infiltrates on chest radiograph, and no evidence of left atrial hypertension/congestive heart failure/fluid overload. The diagnosis of drug-related NCPE relies upon documented exclusion of other causes of NCPE like gastric aspiration, sepsis, trauma, negative pressure pulmonary oedema, etc. We describe two cases (45-year male and 6-year male), who had undergone elective surgery under general anaesthesia. They developed NCPE within 3-5 minutes after administration of ‘neostigmine-glycopyrrolate’ used to reverse residual neuromuscular blockade. Both patients were treated successfully with mechanical ventilatory support, and adjuvant therapy, viz., frusemide, dopamine, steroids. This report emphasizes that this fatal complication may be seen with neostigmine, the pathogenic mechanism remains unknown, and it probably is a drug-related NCPE.

## INTRODUCTION

Pulmonary oedema after general anaesthesia, a rare complication has been earlier reported in literature that can be cardigoenic,[1] non-cardiogenic (NCPE)[2] or negative pressure pulmonary oedema (NPPE).[3–6] Gastric aspiration, sepsis and trauma are well recognized causes of NCPE.[2] Less appreciated is the fact that various drugs, either taken as standard therapy or as an overdose, may precipitate NCPE.[7] Little is known about the mechanisms involved in drug related NCPE.

In this report, we describe two cases of NCPE after administration of neostigmine glycopyrrolate combination used for reversing the residual neuromuscular blockade. Possible causes, therapeutic approach and a brief review of literature has been discussed.

## CASE REPORTS

### Case 1

**Figure 1:**
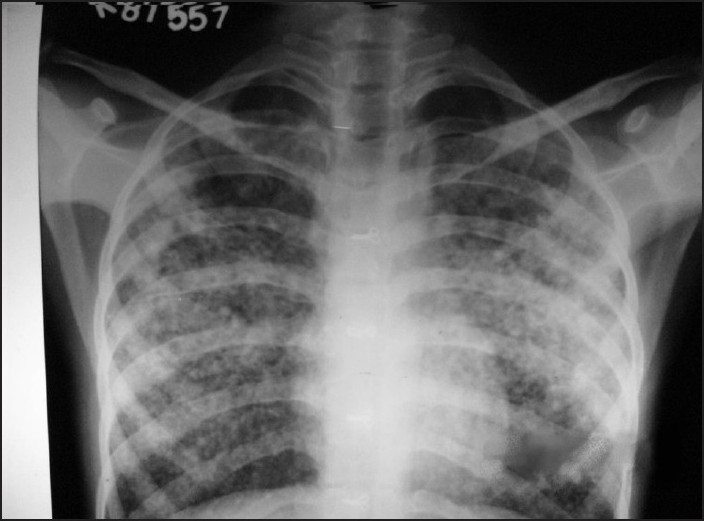
X-ray chest PA view showing pulmonary oedema

A 45-year old male (65 kg) with unremarkable PAE, was taken for excision of haemangioma on lower lip (3 × 3 cm) electively after overnight fasting. He was premedicated with midazolam (2 mg), ondansetron (4 mg), fentanyl (100 µg), diclofenac (75 mg) and induced with thiopentone (300 mg), suxamethonium (100 mg) followed by intubation with Portex 8.5 mm cuffed ETT and oropharyngeal packing. Maintenance of anaesthesia was on propofol (100 µg/kg/min), N_2_O:O_2_(50:50) and vecuronium. Intraoperative monitoring included NIBP, SpO_2_, ECG which remained in normal range during the surgery that lasted 2 hours and 1500 ml Ringer Lactate was given. In the end after thorough oropharyngeal suctioning and removal of pack, neostigmine 2.5 mg and glycopyrrolate 0.4 mg was given. Within 3-5 min, although patient was fully conscious, moving all four limbs with smooth antigravity movements, he was restless, irritable and indicating with his hands that he had difficulty in breathing inspite of adequate tidal volume and good expiratory flow of air; SpO_2_had fallen to 60-80%, BP 130/80, HR 110/min, with normal ECG on monitor. Suddenly sound of secretions started coming from bag, with fine crepts on chest auscultation. There was no wheeze or urticaria on the body and oral cavity was dry. In endotracheal suctioning copious pink frothy secretions were coming out continuously with SpO_2_decreasing to 40-50%. Immediately i.v. fluids were stopped and patient was propped up and catheterized. He was given frusemide, dexamethasone, hydrocortisone, deriphylline followed by vecuronium and diazepam and taken on manual IPPV with 100% O_2_by Bain circuit. Then BP decreased to 90/60, HR 130/min for which dopamine 5-10 µg/kg/min was started with aim to keep SBP above 100 mmHg. Patient was shifted to surgical ICU and taken on ventilatory support (Critivent ^(R)^) on CMV mode with TV 500 ml, f 15/min and PEEP 7 on 100% O_2_. Chest X-ray showed bilateral diffused infiltrate suggestive of pulmonary oedema [Figure 1]. ABG showed pH 7.29, PO_2_70 mmHg, PCO_2_45 mmHg, HCO_3_21, BE -3, SpO_2_= 94%, suggestive of ARDS. All blood investigations came in normal range. ECG was recorded, cardiologist reference was done and it was diagnosed as non-cardiogenic ARDS. He was electively ventilated overnight with vecuronium and midazolam.

Dopamine was weaned during night and next day morning vecuronium and midazolam were stopped and he was taken on SIMV mode with TV 550, f 15, PEEP 5. Patient was weaned off from the ventilator gradually over 4 hours and extubated. Repeat ABG and CXR were normal. Patient was discharged after 2 days uneventfully.

### Case 2

A 6-year old (15 kg) male child was taken for corneal repair after overnight fasting with unremarkable PAE. He was premedicated with appropriate doses of midazolam, tramadol, glycopyrrolate, ondansetron and diclofenac; induced with thiopentone, suxamethonium and intubated with Portex cuffed ETT I.D. 5 mm. Anaesthesia was maintained with N_2_O: O_2_(50:50), propofol and atracurium. Surgery lasted for 45 minutes and 240 ml isolyte-P was given. Vitals remained normal throughout. At the end of surgery reversal in form of noestigmine 1 mg and glycopyrrolate 0.2 mg was given. Within three minutes, patient was fully conscious, opening eyes, had spontaneous respiration with adequate tidal volume, having good muscle power and not tolerating the tube so he was extubated. During that time SpO_2_couldn’t be recorded because patient was moving vigorously. Even after extubation he was irritable, crying and not allowing to keep mask on the face for oxygenation. BP was 100/60, HR 130/min, SpO_2_70%, normal ECG, auscultation of chest showed crepts. Immediately propofol and atracurium was given and on laryngoscopy, frothy secretions were seen at the laryngeal inlet. He was reintuabted with no. 5 cuffed ETT and ventilated with 100% O_2_by Bain circuit. Copious pink frothy secretions coming out through the tube were suctioned frequently. Patient was propped up, catheterized and given steroids, deriphylline and frusemide. Over one hour of ventilation, secretions gradually declined and SpO_2_reached to 95-98%. He was allowed for spontaneous reversal, extubated after one hour and shifted to pediatric ICU.

## DISCUSSION

Pulmonary oedema can reflect either an increase in the net hydrostatic pressure across the pulmonary capillaries called hydrostatic pulmonary oedema that can be cardigoenic[1] or due to fluid overload or an increase in the permeability of the alveolar-capillary membrane called permeability pulmonary oedema (non-cardiogenic pulmonary oedema NCPE/ARDS).[2]

In both of the cases fluid overload was absent and cardiogenic pulmonary oedema has been ruled out because of absence of preexisting heart disease, absolutely normal ECG throughout that was supported by cardiologist’s opinion as NCPE.

In both of the cases aspiration has been ruled out and both did not have sepsis before surgery. According to the “two hit hypotheses”, the bacterial infection preceding the operation may prime the immune cells and the following surgical stress may then trigger sudden massive pulmonary capillary leak leading to ARDS during elective surgery even though the patient’s condition is well controlled.[7]

Negative pressure pulmonary oedema (NPPE) also a form of NCPE, an uncommon problem developed by the occurrence of persistent inspiratory efforts against an obstructed upper airway, creating a markedly negative intra-pleural pressure[8] was ruled out in both cases since both have good exhaled tidal volume.

In both of the present cases, NCPE developed within 3-5 minutes of administration of neostigmine glycopyrrolate combination. In literature, it is mentioned that neostigmine over-dosage may induce cholinergic crisis characterized by excessive salivation, increased bronchial secretion, bradycardia or tachycardia, cardiospasm, bronchospasm and hypotension. Extreme high doses may produce CNS symptoms of agitation, fear or restlessness and death may result from cardiac arrest or respiratory paralysis and pulmonary oedema. Over-dosage are most likely to occur during treatment of myasthenia gravis.[9] In our cases dose used was 0.06 mg/kg ruling out above mechanism of lung injury.

In a recent study on rats, it is reported that neostigmine given in the absence of neuromuscular (NM) block or when given after recovery from NM block, it evokes a partial upper airway obstruction by decreasing skeletal upper airway dilator muscle activity which may generate NPPE. However, human studies will be required to evaluate the clinical relevance of above findings.[10]

NCPE is clearly associated with several agents that have vastly different pharmacological functions (few of them are Methadone, Naloxone, Tocolytics, Salicylates, Hydrochlorthiazide, Protamine, Recombinant interleukin-2, Amiodarone, Bleomycin, insulin, streptokinase, lidocaine, and intrathecal methotrexate).[11] Pulmonary oedema has also been reported due to administration of propofol,[12] hydrochlorthiazide,[13] ondansetron.[14]

Diagnosing drug induced NCPE is actually an exercise of exclusion, as there is no diagnostic test available. It is related to the time proximity of administration of drugs and pathogenesis involves both a direct cytotoxic insult to the lung epithelial cells and induction of cytokine triggered inflammatory response. By distinction to drug-induced pulmonary pneumonitis that may lead to pulmonary fibrosis, NCPE can be reversed upon prompt recognition, following immediate discontinuation of the offensive drug and start of intensive supportive treatment although fatalities have been reported.[15]

## CONCLUSION

We faced two cases of unanticipated occurrence of pulmonary oedema after neostigmine administration at the end of general anaesthesia, which were successfully treated with mechanical ventilation, diuretics, inotropes and steroids. Although, we could not find any drug induced NCPE reported after neostigmine in literature or Internet search; in our opinion, the cause of this pulmonary oedema was drug-induced NCPE.
